# DNA:RNA hybrid G-quadruplex formation upstream of transcription start site

**DOI:** 10.1038/s41598-020-64117-x

**Published:** 2020-05-04

**Authors:** Jia-yu Zhang, Ye Xia, Yu-hua Hao, Zheng Tan

**Affiliations:** 1 State Key Laboratory of Membrane Biology, Institute of Zoology, Chinese Academy of Sciences (CAS), Beijing, 100101 P. R. China; 2 CAS Key Laboratory for Biomedical Effects of Nanomaterials and Nanosafety, Multidisciplinary Research Division, Institute of High Energy Physics, Chinese Academy of Sciences (CAS), Beijing, 100049 P. R. China; 3 Center for Healthy Aging, Changzhi Medical College, Changzhi, 046000 Shanxi P. R. China

**Keywords:** DNA, DNA

## Abstract

Bioinformatic analysis reveals an enrichment of putative DNA:RNA hybrid G-quadruplex-forming sequences (PHQS) on both sides of the transcription start sites (TSSs) in the genome of warm-blooded animals, suggesting a positive selection of PHQSs in evolution and functional role of DNA:RNA hybrid G-quadruplexes (HQs) in transcription. The formation of HQs downstream of TSS in transcribed DNA has been documented under *in vitro* conditions; however, it is still not known if such HQs can form at the upstream side of TSSs. In this study, we report that such HQs can form in transcription in DNA with two to three guanine tracts if RNA carrying the required number of G-tracts is supplied. We also show that the formation of such HQs is dependent on the negative supercoiling generated by RNA polymerases. These results suggest that HQs may also form at the upstream side of TSSs *in vivo* and play a role in transcription since the two requirements are satisfied in cells.

## Introduction

Nucleic acid G-quadruplexes have attracted intense attention owing to their potential role in physiology and their proven existence in cells^1–3^. A G-quadruplex forms when four G-tracts are located either in one nucleic acid molecule (intermolecular G-quadruplex) or in several ones (intermolecular G-quadruplex)^4^. A few years ago, we reported that a new category of intermolecular G-quadruplex, named DNA:RNA hybrid G-quadruplex (HQ), can form in transcription when two to three G-tracts are present on the non-template DNA strand at the downstream side of a transcription start site (TSS) under *in vitro* conditions^5,6^ and in living bacteria cells^2^. Although fewer than four G-tracts are on the DNA strand in these cases, extra G-tracts are supplied from the RNA transcripts to form an HQ. Such putative HQ-forming sequences (PHQSs) are enriched at the upstream side of TSSs as those at the downstream side in similar frequency in animal genes^7^. For this reason, we assume that HQs may also form upstream of TSSs although such an assumption has not been tested.

In this work, we attempt to examine whether a PHQS motif at the upstream side of a TSS can form HQ when RNA transcripts can supply the required extra G-tracts. We used the T7 transcription model in both a linear and plasmid DNA that carried two or three G4 tracts at both the upstream and downstream sides of a TSS, respectively. The formation of HQ in the DNA was detected by electrophoresis mobility shift assay (EMSA), photo-cross-linking, and transcription termination. Our results show that HQ formed as expected upstream of TSS. Moreover, we found that the formation of HQ was promoted by DNA supercoiling generated in transcription.

## Results

### HQ formation in linear double-stranded DNA (dsDNA)

**Figure 1:**
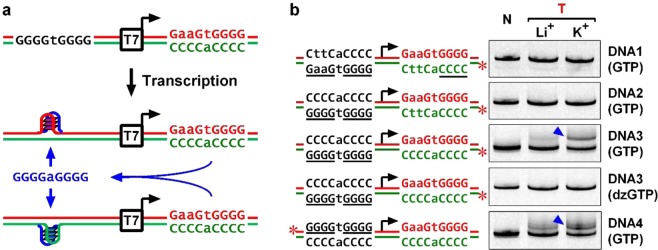
HQ formation in dsDNA upstream of a T7 promoter. (**a**) Scheme of HQ formation in a transcribed DNA. (**b**) Detection of HQ formation in four DNAs using native gel electrophoresis. HQ formation is shown by the extra DNA band (blue arrowhead) migrating behind the original DNA. N, No transcription. T, Transcribed. Asterisk indicates a fluorescein label at the 5’ end.

To test the formation of HQ in linear dsDNA, we arranged a G_4_TG_4_ motif on each side of a T7 promoter (Fig. 1a). Transcription of the DNA by T7 RNA polymerase (RNAP) would produce RNA transcripts carrying a G_4_TG_4_ motif that was expected to join the G_4_TG_4_ motif at the upstream side of the T7 promoter on the DNA to form an HQ. Potential HQ formation downstream of the TSS was prevented by mutations at the non-template strand. The DNA (DNA3) and a few DNA derivatives (DNA1, DNA2, DNA4) were transcribed with T7 RNAP and the formation of HQ in these DNAs was examined by native gel electrophoresis in which a DNA bearing an HQ would display a slower migration than the corresponding DNA without an HQ^1^.

In DNA1, there was one G4 tract at the upstream side and one C4 tract at the downstream side. The transcription of the C4 tract produced RNA transcripts bearing one G4 motif. Because the participation of one G-tract from DNA and one G4 tract from RNA did not satisfy the requirement of four G-tracts, the formation of G-quadruplex was not detected (Fig. 1b). In the DNA2, there were two G4 tracts instead of one at the upstream side of the T7 promoter. Since an RNA transcript could only supply one G4 tract, there was also no sign of HQ formation in this DNA. However, when we added one more C4 tracts at the downstream side of the T7 promoter such that an RNA transcript could provide two G4 tracts; HQ was detected in the DNA3 as indicated by an extra DNA band (blue arrowhead) behind the original DNA.

The formation of a G-quadruplex involves the participation of the N7 of guanine. When this N7 nitrogen in the RNA was replaced by a carbon by substitute the normal GTP with 7-deaza-GTP (dzGTP) during transcription, the extra band disappeared. The DNA4 was similar to DNA3, but having the G_4_TG_4_ motif placed on the non-template instead of on the template strand. HQ formation was also detected, indicating that HQ formation was not strand-dependent. This behavior was in agreement with an un-biased distribution of PHQS motifs in the two DNA strand upstream of TSSs of animal genes^2^. In DNA3 and DNA4, the formation of HQ was much more obvious in K^+^ than in Li^+^ solution because the former is a more effective stabilizer of G-quadruplexes^3–5^.

### HQ formation involves both RNA and DNA

**Figure 2:**
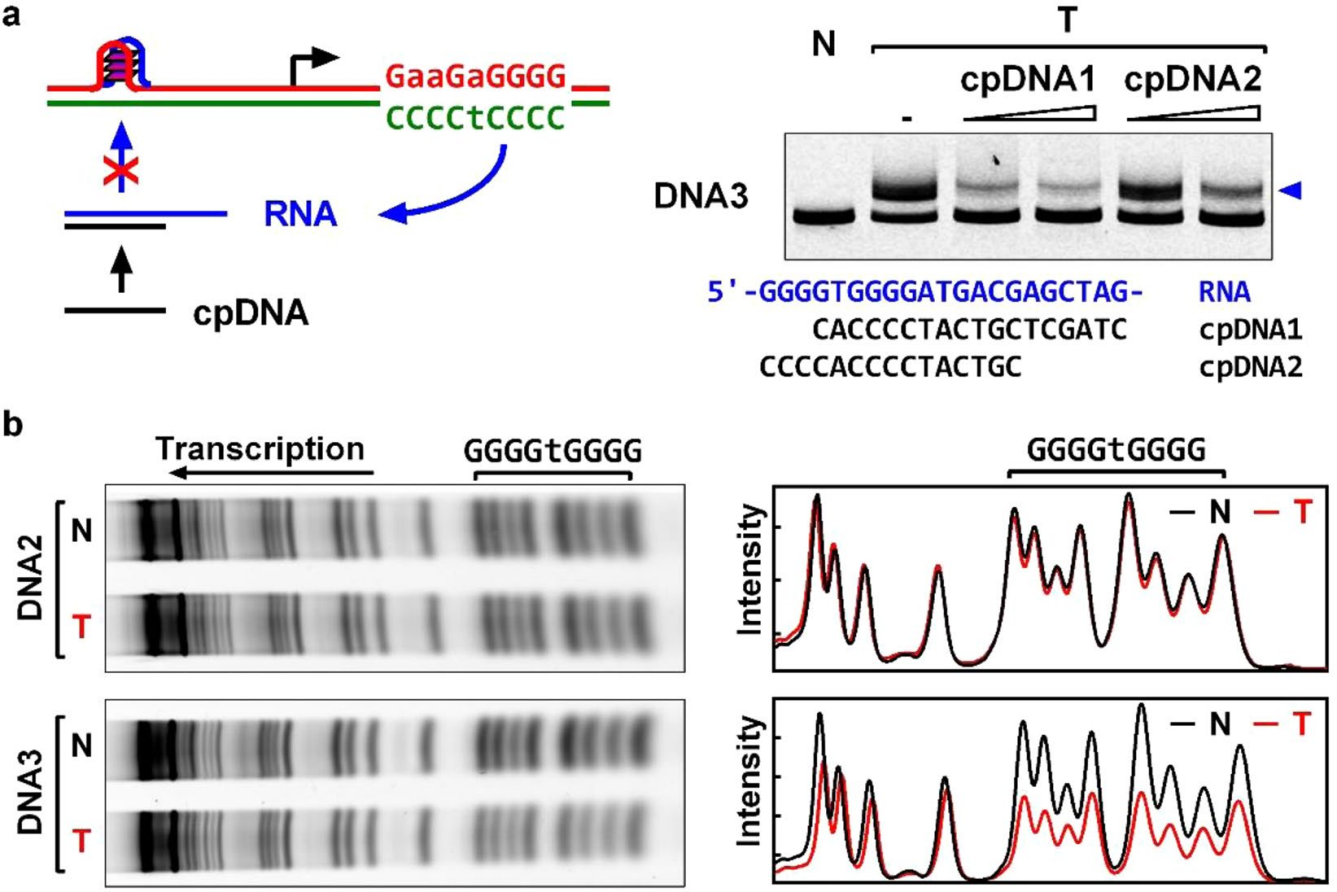
Participation of RNA and DNA in HQ formation upstream of a T7 promoter. (**a**) Native gel electrophoresis showing that HQ formation (blue arrowhead) upstream of TSS required RNA and did not occur when RNA transcripts were captured by complementary DNA oligonucleotides. (**b**) HQ formation involved the G-tracts in DNA upstream of TSS that were protected from cleavage in DMS footprinting. N, No transcription. T, Transcribed.

The dzGTP replacement assay suggested that the formation of HQ involved the participation of RNA transcripts as expected. To verify the involvement of RNA transcripts, we carried out a transcription with DNA3 in the presence of two DNA oligonucleotides complementary to a region of the RNA transcript (Fig. 2a). The complementary DNAs (cpDNA) were intended to capture the RNA transcripts, preventing them from forming HQs with the DNA. As is shown in Fig. 2a (right panel), cpDNA1 severely suppressed the formation of HQ. The suppression of HQ formation became less efficient when a shorter cpDNA2 was used to reduce the intended competition.

On the other hand, we verified the involvement of DNA in the HQ formation by DMS footprinting in which the formation of G-quadruplex will protect the G-tracts from chemical cleavage^6^. As is indicated in Fig. 2b, the two G4 tracts in the G_4_TG_4_ motif of the transcribed DNA were efficiently protected, showing a much lower cleavage signal than those in the un-transcribed DNA. In DNA2 in which no HQ was detected (Fig. 1b), the cleavage signal was identical in both the transcribed and un-transcribed DNA (Fig. 2b).

### HQ formation involves a joint participation of RNA and DNA

We further conducted a photo-cross-linking experiment to demonstrate that the formation of HQ was the result of the simultaneous participation of RNA and DNA. In this assay, transcription was carried out in the presence of a tri-functional compound, sulfosuccinimidyl-2-[6-(biotinamido)-2-(p-azidobenzamido)hexanoamido]ethyl-1,3’-dithiopropionate (SBED)-GMP and the samples were irradiated with UV-light after transcription. Because the SBED-GMP has a phenyl azide group that can react with a primary amine in adenine, guanine, and cytosine under ultraviolet light, the UV irradiation catalyzed a cross-linking between the SBED-GMP incorporated into RNA and the DNA. The cross-linking was then detected in a primer extension assay by extension termination at the cross-linking sites^7,8^.

**Figure 3:**
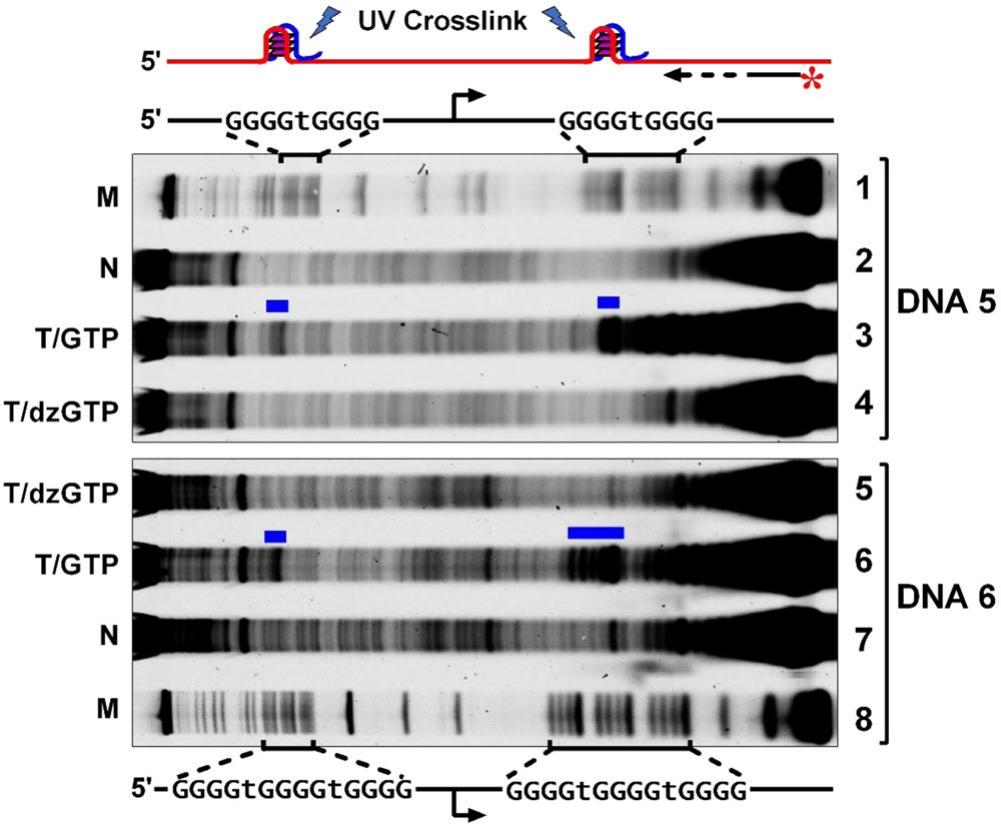
HQ formation upstream of a T7 promoter involved joint participation of RNA and DNA. SBED-GMP was incorporated into RNA during transcription. RNA retained in an HQ was cross-linked with the DNA upon irradiation by UV light. Cross-linking sites were detected by primer extension that terminated at the cross-linking site (blue bar). Asterisk indicates a fluorescein label at the 5’ end. M, Marker. N, No transcription. T, Transcribed. From top to bottom, lanes 1 to 4 represent DNA5; lanes 5 to 8 represent DNA6.

We used two DNAs (DNA5 and DNA6) that carried a G_4_TG_4_ or G_4_TG_4_TG_4_ motif at both sides of the T7 promoter (Fig. 3). In the DNA5, we can see that cross-linking occurred at the G_4_TG_4_ motif upstream of the T7 promoter. In the DNA6 in which a G_4_TG_4_TG_4_ motif was used to facilitate the formation of HQ with more G4 tracts, a stronger signal of cross-linking was seen at the G_4_TG_4_TG_4_ motif upstream of the T7 promoter. In both cases, no cross-linking was detected when the transcriptions were performed using dzGTP instead of GTP to abolish the formation of HQ.

### HQ formation depends on negative supercoiling

**Figure 4:**
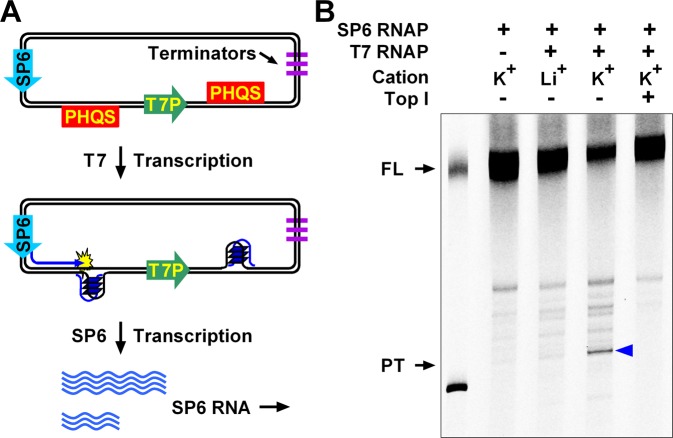
HQ formation upstream of a T7 promoter in plasmid detected by RNA polymerase arrest. (**a**) The plasmid was first transcribed with T7 RNAP to induce the formation of HQ between the SP6 and T7 promoter. Then the plasmid was transcribed by SP6 RNAP to produce fluorescently labeled RNA. The T7 RNAP was captured with an excess amount of competitive DNA to prevent further T7 transcription. SP6 transcripts were resolved by denaturing polyacrylamide gel electrophoresis. (**b**) HQ formation between the SP6 and T7 promoter caused premature termination of SP6 transcription (blue arrowhead). FL, Full-length transcript. PT, prematurely terminated transcript. The two bands in lane 1 are the marker transcripts of the two linearized plasmids, approximately representing the size of the prematurely terminated transcripts and the full-length transcripts. The bottom marker was shorter than the distance between the SP6 promoter and PHQS by 19 nucleotides.

To test HQ formation in a more physiologically relevant situation, we transferred the T7 transcription model into a plasmid. In addition to a T7 promoter with two PHQS (G_4_TG_4_TG_4_) motifs on both sides, the plasmid also had an SP6 promoter upstream of the three elements (Figure 4a). The plasmid was first transcribed by T7 RNAP to induce a formation of HQ between the SP6 and T7 promoters. Then transcription with the SP6 promoter was subsequently performed using fluorescein-12-UTP. The fluorescently labeled RNA transcripts were resolved by denaturing electrophoresis and HQ formation was determined by transcription premature termination at the HQ^9^.

As is shown in Fig. 4b, transcription initiated from the SP6 promoter was partially terminated by the HQ induced by the T7 transcription, producing prematurely terminated (PT) transcripts (lane4, arrowhead). The termination was more obvious in K^+^ (lane 4) than in Li^+^ (lane 3) solution, implying a stronger formation of HQ in the K^+^ than in the Li^+^ solution, which was in agreement with the results obtained with linear dsDNA (Figure 1). Previous studies have demonstrated that the formation of G-quadruplexes is sensitive to the supercoiling state of a plasmid^9^. A transcription using a relaxed plasmid in the presence of topoisomerase I (Top1) did not yield prematurely terminated RNA transcripts (lane 5). This result indicated the formation of HQ is also facilitated by negative supercoiling.

## Discussion

Our work demonstrates that a PHQS motif upstream of a TSS can form an HQ when G-rich RNA transcripts are supplied. The formation of HQ only occurs when the RNA transcripts can supply the required number of G-tracts to a PHQS motif in a DNA strand. Transcription with a mutated template DNA strand or using dzGTP in place of GTP abolished the capability of RNAs to participate in G-quadruplex formation and, as a result, prevented the formation of the HQ. In addition, trapping the RNA transcripts by a C-rich complementary DNA inhibited the HQ formation.

In transcription, a moving RNAP produces a negative supercoiling torsion wave that transmits upwards behind the RNAP^10^, which is able to create single-stranded patches of DNA^11^. This DNA strand separation provides a chance for a G-quadruplex to form. In the same T7 transcription model, transcription with T7 RNAP has been reported to induce a robust formation of intramolecular G-quadruplexes at the upstream side of a TSS by the upward transmission of negative supercoiling torsion wave^12,13^. The failure to observe the formation of HQ when supercoiling in the plasmid was dispatched with TopI indicated that the formation of HQ was driven by the same mechanism. For a free linear dsDNA, transcription-generated supercoiling can be released by rotating the DNA around the helical axis^14^. The formation of HQ in the linear dsDNA in our experiments might be attributed to the restriction of DNA rotation imposed by the high solution viscosity in the presence of PEG.

PHQS motifs with two to three G-tracts are several times more abundant than the canonical PQS motifs in the human genome. Our results show that such PHQS motifs may have a chance to form HQs when G-rich RNAs are available in proximity. In human genes, PHQS motifs are remarkably enriched near TSSs at both the upstream and downstream sides^2^. G-rich RNA transcripts originated from the downstream sides may supply the required G-tracts for HQs to form at the upstream side of TSSs. This assumption explains the enrichment of PHQS motifs at the upstream side of TSSs in animal genes and predicts a functional role of such HQs in transcription.

## Methods

### Oligonucleotides and double-stranded DNA

Oligonucleotides (Table 1) were synthesized by Sangon Biotech (Shanghai, China). Double-stranded DNA was prepared with two complementary or partially complementary oligonucleotides annealing at a rate of 0.03 °C/s from 95 °C to 25 °C in a buffer containing 10 mM LiAsO_2_(CH3)_2_, pH 7.9, 50 mM LiCl. Double-stranded DNA for photo-cross-linking was prepared by overlap polymerase chain reaction (PCR) as we previously described^1^.

### *In vitro* transcription

*In vitro* transcription was performed as previously described with minor modification^1^. 0.25 μM DNA was transcribed with 200 U T7 RNA polymerase (Thermo Scientific, EP0113) in 25 μL of transcription buffer containing 40 mM HEPES, pH 7.9, 40% PEG 200, 50 mM KCl or LiCl, 10 mM MgCl_2_, 10 mM DDT, 2 mM spermidine, and 1 mM NTP set (ATP, CTP, UTP, GTP) (Thermo Scientific, R0481) at 37 °C for 1 h. In some experiments, GTP was replaced by 1 mM 7-deaza-GTP (dzGTP) and 0.4 mM GMP. Post transcription digestion with RNase and proteinase K was carried out as described^1,4,15^. The same volume of digestion buffer containing 10 mM Tris-HCl pH 7.9, 40% PEG 200, 50 mM KCl or LiCl and 1 μg RNase A (Thermo Scientific, EN0531) was mixed with the samples and incubated at 37 °C for 0.5 h followed by treated with 1 μg Proteinase K (Takara, 9034) for another 0.5 h. the samples were resolved on 12% native polyacrylamide gel.

### Photo-cross-linking

DNA was transcribed in 100 μl transcription buffer as aforementioned except that DTT was excluded and 1 mM SBED-GMP^8^ was added to the transcription buffer. After transcription at 37 °C for 1 hour, the samples were irradiated and subjected to primer extension as the previous studies^1,4^. DNA was purified by Wizard SV Gel and PCR Clean-Up System (Promega) followed by primer extension with Deep Vent (exo-) (NEB, M0259) and FAM-5’-TGACAGCGATGCGTAGAATCGCTAG. The G ladder was obtained by extension of non-transcribed DNA in the presence of Acy-CTP (NEB, N0460).

### DMS foot printing

DMS footing was carried out as previously described^1,15^. In brief, 4 μl 10% DMS was added to 200 μl transcription buffer and incubated at 37 °C for 6 min. 75 μl stop buffer containing 200 μg fish sperm DNA and 0.95 M β-Mercaptoethanol was added to terminate the reaction. After phenol/chloroform extraction and ethanol precipitation, the precipitate was dissolved in 100 μl of a 10% piperidine solution and incubated at 90 °C for 30 minutes. Chloroform extraction and ethanol precipitation were performed again so that the samples were dissolved in 40 μl ddH2O and subjected to denaturing gel electrophoresis for imaging on Typhoon 9400 (GE Healthcare).

### Gel electrophoresis

Samples resolved on the native polyacrylamide gel containing 40% (w/v) PEG 200 and 75 mM KCl were electrophoresed at 4 °C, 12 V/cm. The denaturing polyacrylamide gel electrophoresis was carried out at RT, 25 V/cm^15^. The images were obtained on Typhoon 9400 and analyzed by ImageQuant software (version 5.2, GE Healthcare).

### Plasmid construction

The plasmid was constructed by inserting a DNA fragment with G-tracts on both sides of a T7 promoter between an SP6 promoter and a transcription terminator as described^9^.

### Polymerase arrest assay

Polymerase arrest assay was carried out as previously described^9^. Briefly, the plasmid was transcribed by T7 RNA polymerase in 25 μl transcription system at 37 °C for 1 hour. The system was almost the same as the *in vitro* transcription one except that 0.1 U inorganic pyrophosphatase (Thermo Scientific, EF0221) and 1 U/μL RiboLock RNase inhibitor (Thermo Scientific, EO0381) were included while PEG 200 was excluded. After the transcription was terminated by 2 mM competitive DNA, 20 U SP6 RNA polymerase (Thermo Scientific, EP0131) and a final concentration of 0.2 mM fluorescein-12-UTP (Roche, 11427857910) were added and incubated at 37 °C for another 30 min. The samples were treated with 0.4 U DNase I at 37 °C for 15 min and extracted by phenol-chloroform. 8% denaturing polyacrylamide gel was applied to examine the RNA transcript.

**Table 1:** Sequence of oligonucleotides.

DNA No.	Sequence (5’ to 3’)
DNA1	ATGAGAGTACTTCACCCCTGGTCATTGTCATCACTAGATAATACGACTCACTATAGAAGTGGGGATGACGAGCTAGCGATTCTACGCATCGCTGTCA
DNA2	ATGAGAGTACCCCACCCCTGGTCATTGTCATCACTAGATAATACGACTCACTATAGAAGTGGGGATGACGAGCTAGCGATTCTACGCATCGCTGTCA
DNA3	ATGAGAGTACCCCACCCCTGGTCATTGTCATCACTAGATAATACGACTCACTATAGAAGTGGGGATGACGAGCTAGCGATTCTACGCATCGCTGTCA
DNA4	ATGAGAGTAGGGGAGGGGTCATCATTGTCATCACTAGATAATACGACTCACTATAGAAGTGGGGATGACGAGCTAGCGATTCTACGCATCGCTGTCA
DNA5	AAGTTTGCACGCCTGCCGTTCGACGATTATGAGAGTAGGGGTGGGGTCATCATTGTCATCACTAGATAATACGACTCACTATAGGGGTGGGGATGACGAGCTAGCGATTCTACGCATCGCTGTCA
DNA6	AAGTTTGCACGCCTGCCGTTCGACGATTATGAGAGTAGGGGTGGGGTGGGGCATCATTGTCATCACTAGATAATACGACTCACTATAAGGGTGGGGAGGGGATGACGAGCTAGCGATTCTACGCATCGCTGTCA
Extension primer	TGACAGCGATGCGTAGAATCGCTAG
cpDNA1	CTAGCTCGTCATCCCCAC
cpDNA2	CGTCATCCCCACCCC
Competitive DNA	GAAATTAATACGACTCACTATA

## Supplementary information

Supplementary information.
