# Supplementary material for: DNA:RNA hybrid G-quadruplex formation upstream of transcription start site
Source: Sci Rep. 2020 May 4;10:7429. doi: 10.1038/s41598-020-64117-x (PMC7198591; doi:10.1038/s41598-020-64117-x)
Supplement: Supplementary file 1 — Supplementary information. [file 41598_2020_64117_MOESM1_ESM.docx]

Supporting Information

**DNA:RNA hybrid G-quadruplex formation upstream of transcription start site**

# Jia-yu Zhang1,2, Ye Xia1, Yu-hua Hao1, and Zheng Tan1,3,*

1 State Key Laboratory of Membrane Biology, Institute of Zoology, Chinese Academy of Sciences (CAS), Beijing 100101, P. R. China

2CAS Key Laboratory for Biomedical Effects of Nanomaterials and Nanosafety, Multidisciplinary Research Division, Institute of High Energy Physics, Chinese Academy of Sciences (CAS), Beijing 100049, P. R. China

3Center for Healthy Aging, Changzhi Medical College, Changzhi 046000, Shanxi, P. R. China

*[corresponding.](mailto:corresponding.author@email.example) [z.tan@ioz.ac.cn](mailto:z.tan@ioz.ac.cn)

# ABSTRACT

Bioinformatic analysis reveals an enrichment of putative DNA:RNA hybrid G-quadruplex-forming sequences (PHQS) on both sides of the transcription start sites (TSSs) in the genome of warm-blooded animals, suggesting a positive selection of PHQSs in evolution and functional role of DNA:RNA hybrid G-quadruplexes (HQs) in transcription. The formation of HQs downstream of TSS in transcribed DNA has been documented under in vitro conditions; however, it is still not known if such HQs can form at the upstream side of TSSs. In this study, we report that such HQs can form in transcription in DNA with two to three guanine tracts if RNA carrying the required number of G-tracts is supplied. We also show that the formation of such HQs is dependent on the negative supercoiling generated by RNA polymerases. These results suggest that HQs may also form at the upstream side of TSSs in vivo and play a role in transcription since the two requirements are satisfied in cells.





Figure S1. The full-length gel of part of Figure 1.





Figure S2. The full-length gel of the other part of Figure 1.





Figure S3. The full-length gel of Figure 2a.





Figure S4. The full-length gel of Figure 2b.





Figure S5. The full-length gel of Figure 3.





Figure S6. The full-length gel of Figure 4b.
